# Synergic trial secondary outcomes results on gait and falls

**DOI:** 10.1093/geroni/igaf122.2643

**Published:** 2025-12-31

**Authors:** Frederico Faria, Surim Son, Teresa Liu-Ambrose, Quincy Almeida, Laura Middleton, Louis Bherer, Manuel Montero Odasso

**Affiliations:** Western University Lawson Health Research Institute, London, Ontario, Canada; Western University, London, Ontario, Canada; The University of British Columbia, Vancouver, British Columbia, Canada; Wilfrid Laurier University, Waterloo, Ontario, Canada; University of Waterloo, Waterloo, Ontario, Canada; Université de Montréal, Montreal, Quebec, Canada; University of Western Ontario, London, Ontario, Canada

## Abstract

**Background:**

Older adults with Mild Cognitive Impairment (MCI) have higher risk of gait impairments and falls, yet the effects of multimodal interventions, including combinations of exercises with cognitive training, on improving their mobility remain unclear.

**Objectives:**

To investigate the synergistic effects of aerobic-resistance exercise combined with cognitive training, with or without vitamin D supplementation, on gait performance and fall risk in older adults with Mild Cognitive Impairment (MCI).

**Methods:**

The effect of 20 weeks of aerobic-resistance exercise, cognitive training, and vitamin D supplementation (10,000 IU 3x/week) on gait and falls in older adults with MCI was evaluated in the SYNERGIC Trial, using a fractional factorial design.

**Results:**

Among 161 participants, exercise-based arms (n = 4) improved gait speed (+7.5 cm/s, p < 0.001) and reduced injurious falls by 62% (IRR=0.38, p = 0.04). Exercise plus cognitive training showed the greatest gains in gait speed and reduced injurious falls (IRR=0.13, p = 0.05). Vitamin D did not enhance outcomes and increased gait instability.

**Conclusion:**

Aerobic-resistance exercise combined with cognitive training improves gait performance and decreases the risk of injurious falls in older adults with MCI. The addition of vitamin D did not produce benefits.

